# Comparison of the Fermentation Activities and Volatile Flavor Profiles of Chinese Rice Wine Fermented Using an Artificial Starter, a Traditional JIUYAO and a Commercial Starter

**DOI:** 10.3389/fmicb.2021.716281

**Published:** 2021-09-20

**Authors:** Chen Chen, Zheng Liu, Wenya Zhou, Huaixiang Tian, Juan Huang, Haibin Yuan, Haiyan Yu

**Affiliations:** Department of Food Science and Technology, Shanghai Institute of Technology, Shanghai, China

**Keywords:** Chinese rice wine, artificial starter, JIUYAO, flavor profiles, response surface methodology

## Abstract

In this study, an artificial starter culture was prepared using the core microbial species of JIUYAO to produce Chinese rice wine (CRW). The fermentation activity and flavor profiles of CRW samples fermented with traditional JIUYAO, a commercial starter culture, and our artificial starter culture were compared. The optimal protectant combination for lyophilization of the artificial starter was established as 15.09% skim milk, 4.45% polyethylene glycol, 1.96% sodium glutamate, and 11.81% maltodextrin. A comparative analysis revealed that the ethanol content of the three CRW samples was similar. The total acid content of the CRW sample fermented with the artificial starter (7.10 g/L) was close to that of the sample fermented with JIUYAO (7.35 g/L), but higher than that of the sample fermented with the commercial starter (5.40 g/L). An electronic nose analysis revealed that the olfactory fingerprints of the CRW samples fermented with JIUYAO and the artificial starter resembled each other. For both above mentioned samples, the flavor profiles determined by gas chromatography–mass spectrometry indicated some differences in the variety and content of the aroma compounds, but the key odorants (odor activity values ≥1), such as isoamyl acetate, ethyl acetate, phenyl alcohol, and isoamyl alcohol, were similar.

## Introduction

Chinese rice wine (CRW), which has a high nutritional value and distinctive flavor, has been consumed for centuries (Xu et al., 2015; Huang Z. R. et al., 2018). CRW includes many renowned types, such as Shaoxing, Jimo, and Fujian rice wine (Jiao et al., 2017). The best-known CRW, Shaoxing rice wine, is generally produced using glutinous rice, traditional JIUYAO, and wheat *qu* (Chen et al., 2013). JIUYAO is a mixed starter culture that mainly includes bacteria, molds, and yeast and is responsible for the starch saccharification and fermentation in Shaoxing rice wine brewing (Liu et al., 2018). The microorganisms in JIUYAO are believed to play a crucial role in the fermentation activity and unique flavor formation of Shaoxing rice wine (Xie et al., 2007). Zhao et al. (2020) revealed that *Proteobacteria* and *Firmicutes* are the dominant bacterial phyla in JIUYAO. Our previous study (Chen et al., 2020) found that only five core species, two from the *Weissella* genus and one each from the *Pediococcus*, *Saccharomycopsis*, and *Rhizopus* genera, play a key role in the flavor formation and fermentation activity of CRW.

Currently, commercial starters that mainly include yeast and *Rhizopus* are widely used in the industrial brewing of CRW (Ferrer-Gallego et al., 2014). Although the fermentation period of CRW brewed with commercial starters is comparable to that of CRW brewed with traditional JIUYAO (Huang et al., 2019), the flavor of the former is inferior due to the limited microbial variety in the commercial starters (Liu et al., 2019). However, as traditional JIUYAO is usually handcrafted, its limitation is the instability of the resulting CRW’s quality and flavor profiles across batches (Zhao and Zhang, 2010). Therefore, it would be of great significance to develop an artificial starter culture as a replacement for traditional JIUYAO that could yield CRW batches of high quality and uniform flavor.

A microbial consortium selected as a starter culture should be able to withstand subsequent fermentation. Therefore, lyophilization techniques and low temperatures (refrigeration or freezing) are usually used to stabilize the starter culture (Abadias et al., 2001; Pradelles et al., 2009). Lyophilization is a simple technique to maintain a high number of viable microorganisms in a starter culture inoculum in powder form, as it involves few procedural steps. However, this preservation process can cause cell damage or lead to cell death (Ale et al., 2015). Cell mortality during lyophilization can be minimized by optimizing freeze-drying conditions and using lyoprotectants (Cerrutti et al., 2000; Hubálek, 2003), such as proteins, skim milk, sugars, and other biopolymers (Coulibaly et al., 2016). Although freeze-dried bacterial powder with protectants has been used as a starter culture (Chen H. et al., 2015), its use in place of traditional JIUYAO for CRW brewing has not been documented.

In this study, an artificial starter culture was prepared by combining the core microorganisms from traditional JIUYAO that contribute to the unique flavor of CRW, and then freeze-drying them to powder form using a lyoprotectant combination optimized through a response surface optimization experiment. The fermentation activity and flavor profile of the CRW samples fermented with this artificial starter were compared with those of samples fermented with traditional JIUYAO and a commercial starter culture. The use of such an artificial starter can improve and standardize the fermentation activity and aroma quality of CRW, and further promote the industrial production of traditional CRW.

## Materials and Methods

### JIUYAO, Starter Culture and Lyoprotectants

JIUYAO samples were obtained from Zhejiang Tapai Shaoxing Rice Wine Co., Ltd., Shaoxing city, Zhejiang province, China. The samples were refrigerated at 4°C before being transported to the laboratory. A commercial starter culture composed of *Saccharomyces cerevisiae* and *Rhizopus oryzae* was purchased from a commercial yeast and yeast extract manufacturer in China. Five microbial species, namely *Pediococcus pentosaceus* (CCTCC M 2019323), *Weissella cibaria* (BNCC206838), *W. confusa* (CICC23465), *Saccharomycopsis fibuligera* (CCTCC M 2019324), and *R. arrhizus* (CCTCC M 2019325), were selected and isolated as the core functional microbes from the JIUYAO samples, due to their key role in the fermentation activity and flavor formation of CRW, as determined in our previous study (Chen et al., 2020). To ensure that the samples had the ability to produce ethanol, *S. cerevisiae* (NKCCMR NK3. 00156) was also added to the artificial starter (Chen et al., 2020). The isolated strains were stored at −80°C and used after reactivation by successive subcultures in steamed rice. Briefly, for activation, *W. cibaria*, *W. confusa*, and *P. pentosaceus* (2% vol/vol) were inoculated into MRS broth (Merck, Darmstadt, Germany) and incubated at 37°C for 18 h, whereas *S. fibuligera*, *S. cerevisiae*, and *R. arrhizus* (2% vol/vol) were inoculated in MEB liquid medium (Merck, Darmstadt, Germany) and incubated at 30°C for 24 h. The inoculum concentrations of the individual core bacterial species were determined according to the amount of JIUYAO added to the CRW, the total number of bacterial and fungal colonies in the JIUYAO, and the abundance of core microbial species in JIUYAO as described in our previous study (Chen et al., 2020). The final concentrations of the core microbes that were combined to form the artificial starter culture were *P. pentosaceus* (8.6 × 10^3^ CFU/g), *S. fibuligera* (9.6 × 10^3^ CFU/g), *R. arrhizus* (10.6 × 10^2^CFU/g), *W. cibaria* (2.2 × 10^3^ CFU/g), *W. confuse* (2.2 × 10^3^ CFU/g), and *S. cerevisiae* (2.0 × 10^4^ CFU/g).

For use as lyoprotectants, skim milk was purchased from Fonterra Ltd. (New Zealand), and polyethylene glycol, sodium glutamate, and maltodextrin of food grade were purchased from WanBang Co. Ltd. (Zhengzhou, Henan Province, China).

### Freeze-Drying of the Starter

All of the protectants used in the experiment were dissolved in distilled water to obtain various concentrations. They were sterilized at 115°C for 15 min and stored at 4°C until use.

Different concentrations of the protectant solutions were added to the culture pellets of the core microbes in the ratio 1:1 (vol/vol). After mixing, the samples were pre-frozen at −80°C for 2 h, placed in a vacuum freeze-dryer at −80°C and 0.162 mbar vacuum for 48 h, and finally stored at −20°C until use (Qi et al., 2017).

The lyophilization survival factor (*SF*_*L*_) was calculated following the formula given by Hubálek (2003):


$$SF_{L}=1-[\left(\text{log}CFU/mL_{i\,n\,i\,t\,i\,a\,l}-logCFU/mL_{f\,i\,n\,a\,l}\right)/logCFU/mL_{i\,n\,i\,t\,i\,a\,l}]$$

where CFU/mL_*initial*_ is the number of viable cells before lyophilization, and CFU/mL_*final*_ is the number of viable cells after lyophilization.

### Experimental Design for Optimization of the Lyoprotectant Composition

A lyoprotectant is usually added to the target solution of cells before the freeze-drying process. It adds a matrix around the cells that protects them from drying and freezing and increases their survival ability (Abadias et al., 2001). Ten lyoprotectants were selected and mixed thoroughly with the core microbes, and the survival factors of the microbes were determined as an indicator of their survival in our preliminary study (data not shown). Finally, four lyoprotectants with the best protective effects were selected for our experiment: skim milk, polyethylene glycol, sodium glutamate, and maltodextrin. These selected lyoprotectants were diluted to different concentration gradients (skim milk and maltodextrin: 5, 10, 15, 20 and 25%; polyethylene glycol: 1, 3, 5, 7 and 9%; sodium gluconate: 0.5, 1, 1.5, 2 and 2.5%), and their optimal concentrations were determined based on the cell survival factor.

The lyoprotectant composition was further optimized using a four-factor, three-level Box–Behnken design and three levels of the *N* = 27 test with Y (lyophilization survival rate) as the response value. The factor levels are shown in Table 1. The following polynomial equation was used:


$$\text{Y}={\text{a}}_{0\,}+{\text{a}}_{1}\,\text{A}+{\text{a}}_{2}\,\text{B}+{\text{a}}_{3}\,\text{C}+{\text{a}}_{4}\,\text{D}+{\text{a}}_{12}\,\text{AB}+{\text{a}}_{13}\,\text{AC}+{\text{a}}_{14}\,\text{AD}+{\text{a}}_{23}\,\text{BC}+{\text{a}}_{24}\,\text{BD}+{\text{a}}_{34}\,\text{CD}+{\text{a}}_{11}\,{\text{A}}^{2}+{\text{a}}_{22}\,{\text{B}}^{2}+{\text{a}}_{33}\,{\text{C}}^{2}+{\text{a}}_{44}\,{\text{D}}^{2}$$

**TABLE 1:** Experimental factor levels of the Box–Behnken design.

Factors	Level		
	−1	0	1
A skim milk (%)	10	15	20
B polyethylene glycol (%)	3	5	7
C sodium glutamate (%)	1.5	2	2.5
D maltodextrin (%)	5	10	15

where Y is the predicted response; A, B, C, and D are independent variables representing the concentration of the four protectants skim milk, polyethylene glycol, sodium glutamate, and maltodextrin, respectively; a_0_ is the second-order reaction constant; a_1_, a_2_, a_3_, and a_4_ are the linear coefficients; a_11_, a_22_, a_33_, and a_44_ are the quadratic coefficients; and a_12_, a_13_, a_14_, a_23_, a_24_, and a_34_ are the interaction coefficients (Crowell and Ough, 1979).

### CRW Brewing

The main steps in CRW brewing are rice soaking, steaming, cooling, starter addition, and fermentation. Briefly, glutinous rice (100 g) was soaked in 100 mL water for 12 h at 25°C. After steaming the soaked rice for 20 min and then cooling it to 25–30°C, rice fermentation was initiated by adding JIUYAO, the artificial starter, or the commercial starter at a final concentration of 0.002 g/g steamed rice. The fermentation process was performed at 29°C for 30 h. The acid content, ethanol content, and saccharification power were determined as described in our previous study (Chen et al., 2020). All chemical determination experiments were performed in triplicate.

### Flash GC Electronic Nose Detection

A HERACLES flash GC electronic nose (Alpha M.O.S., Toulouse, France) equipped with an MXT-5 column and an MXT-1701 column was used for the aroma analysis of the CRW samples. This instrument can perform a complete data analysis owing to its integration with classical gas chromatography (GC) functionalities and electronic nose (e-nose) olfactory fingerprint software.

Briefly, 5 mL of each CRW sample was added to a separate 20-mL vial and incubated at 25°C for 30 min. Hydrogen was circulated at a constant flow rate of 1 mL/min, and 5 mL of headspace gas was injected into the GC port at 200°C. The temperature changes in the GC column were as follows: 50°C for 2 s, a 1°C/s ramp to 80°C, and then a 3°C/s ramp to 250°C with a 15 s hold. The temperature of the detector was 260°C, and each sample was analyzed five times.

### Volatile Compound Analysis

The volatile compound profiles of the CRW samples were analyzed using the headspace solid-phase microextraction (HS-SPME)/gas chromatography–mass spectrometry (GC-MS) approach (Yu et al., 2019b). Briefly, 5 mL of each CRW sample was added to 20 μL of internal standard (2-octanol, 410 mg/L) in a 15-mL headspace glass vial. A fiber (50 μm DVB/CAR/PDMS, Supelco Inc., Bellefonte, Pennsylvania, United States) was exposed to the headspace of the glass vial for 50 min at 50°C.

An Agilent 7890 GC instrument was coupled to a 5973C MS detector (Santa Clara, CA, United States). A capillary HP-Innowax column from Agilent Technologies (60 m × 0.25 mm × 0.25 μm) was used to perform the chromatographic separation. After extraction, the fiber was immediately introduced into the GC instrument, and desorption was performed at 250°C for 5 min. The temperature changes were as follows: the initial temperature was maintained at 40°C, increased to 120°C at a rate of 3°C/min, held for 5 min, and then increased to 200°C at a rate of 3°C/min. Helium was used as the carrier gas at a flow rate of 1 mL/min. The transfer line temperature was 250°C. The mass spectrometers were operated in the electron ionization mode at 70 eV, with a scan range of m/z 30–450.

The compounds were identified by comparing their retention indices (RIs) with those reported in the literature and matching their MS spectra with those in the NIST 11 database. The RIs were determined in relation to those of the C_5_–C_30_ alkane standards (Sigma-Aldrich, St. Louis, MO, United States). To evaluate the sensory contributions of the compounds to the flavor of the CRW samples, their odor activity values (OAVs) were obtained, given by the ratio of the compound concentration in the sample to the threshold concentration in water (Van Gemert, 2003).

### Statistical Analysis

XLSTAT version 7.5 (Addinsoft, New York, NY, United States) was used to analyze the GC-MS data. Design Expert software (version 9, Stat-Ease Inc., Minneapolis, MN, United States) was used for the regression and graphical analysis of the experimental data. The optimal values of the four protectants were calculated using response surface methodology. The regression equations of the models were evaluated using the *F*-test for the analysis of variance. A principal component analysis was performed using WinMuster version 1.6.2 (Alpha M.O.S., Toulouse, France). Origin version 9.0 (Origin Lab Inc., Hampton, MS, United States) and SPSS version 19.0 (SPSS Inc., Chicago, IL, United States) were used for further data analysis.

## Results and Discussion

### Effects of Lyoprotectants on the Survival Factor of the Artificial Starter After Freeze-Drying

**FIGURE 1:**
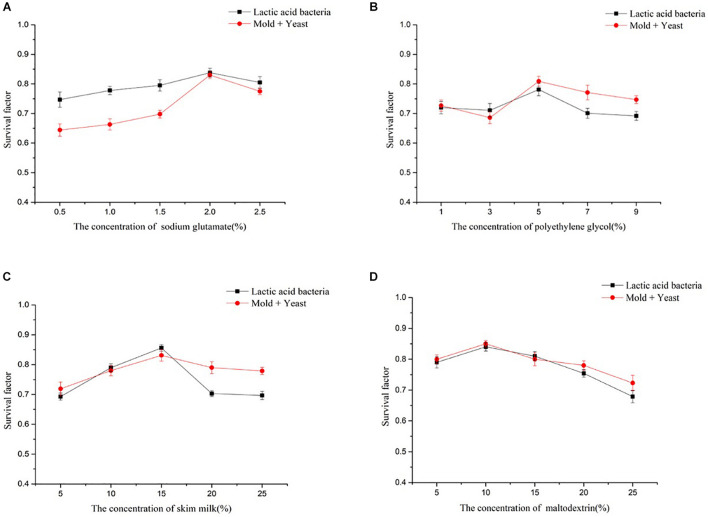
Effects of different concentrations of four lyoprotectants on the survival factor of the artificial starter culture. **(A)** Sodium glutamate, **(B)** polyethylene glycol, **(C)** skim milk, and **(D)** maltodextrin.

Lyophilization has several limitations, such as the formation of ice crystals, altered permeability of the cell membrane, and denaturation and inactivation of sensitive proteins (Yang et al., 2012; Lbg et al., 2020). The role of a lyoprotectant is to prevent these adverse effects. Skim milk, polyethylene glycol, sodium glutamate, and maltodextrin were selected as the protective agents for the artificial starter culture prepared in this study. The results of single-factor experiments indicated that with 15% skim milk, 5% polyethylene glycol, 2% sodium glutamate, or 10% maltodextrin as individual protective agents during freeze-drying, the maximum survival rate of the artificial starter culture was 85.55, 80.94, 83.83, or 85.01%, respectively (Figure 1). The survival factor of the artificial starter gradually improved with increasing concentrations of the protective agents, but after a certain concentration, the survival rate remained unchanged or slowly decreased. This phenomenon was in line with the results reported by Chen et al. (2019), who found that extremely high concentrations of protective agents accelerated the repolymerization of proteins in the cells, resulting in poor survival of the artificial starter.

### Predictive Modeling for the Concentration of Protective Agents

To further improve the survival factor of the microbes in the artificial starter, a predictive model was established for the optimal protectant combination comprising skim milk, polyethylene glycol, sodium glutamate, and maltodextrin. Based on the combination of the Box–Behnken design and the response surface method, an empirical quadratic model was selected to establish the correlation between the independent variables and the response of the survival factor, as follows:


$$\text{Y}=-0.393\,+\,0.036\,\text{A}+\,0.132\,\text{B}+\,0.617\,\text{C}+\,0.028\,\text{D}+\,3.25\,\times \,10^{-4}\,\text{AB}-\,2.40\,\times \,10^{-3}\,\text{AC}+\,1.15\,\times \,10^{-3}\,\text{AD}-\,5.75\,\times \,10^{-3}\,\text{BC}-\,3.725\,\times \,10^{-3}\,\text{BD}+\,2.6\,\times \,10^{-3}\,\text{CD}-1.526\times 10^{-3}\,{\text{A}}^{2}-9.131\times 10^{-3}\,{\text{B}}^{2}-0.149\times 10^{-3}\,{\text{C}}^{2}-1.446\times 10\,{}^{-3}\,{\text{D}}^{2}$$

The *F*-value of the model was 35.22, indicating that this model was significant. The *p*-values of the lack of fit (*p* = 0.3293) and the model (*p* < 0.0001) (Table 2) indicated that the actual corresponding survival factor values exhibited a good fit with this model.

**TABLE 2:** Regression model analysis of variance.

Source	Sum of squares	Degree of freedom	Mean squared	*F*-value	*p*-Value^a^
Model	^0.038^	^14^	2.683 × 10^–3^	^35.22^	< 0.0001^*^^*^^*^
A	2.621 × 10^–3^	1	2.621 × 10^–3^	34.40	< 0.0001^*^^*^^*^
B	6.196 × 10^–3^	1	6.196 × 10^–3^	81.35	< 0.0001^*^^*^^*^
C	6.440 × 10^–3^	1	6.440 × 10^–3^	84.54	< 0.0001^*^^*^^*^
D	1.927 × 10^–3^	1	1.927 × 10^–3^	25.30	0.0002^*^^*^
AB	4.225 × 10^–5^	1	4.225 × 10^–5^	0.55	0.4687
AC	1.440 × 10^–4^	1	1.440 × 10^–4^	1.89	0.1908
AD	3.306 × 10^–3^	1	3.306 × 10^–3^	43.41	< 0.0001^*^^*^^*^
BC	1.322 × 10^–4^	1	1.322 × 10^–4^	1.74	0.2088
BD	5.550 × 10^–3^	1	5.550 × 10^–3^	72.87	< 0.0001^*^^*^^*^
CD	1.690 × 10^–4^	1	1.690 × 10^–4^	2.22	0.1585
A^2^	9.441 × 10^–3^	1	9.441 × 10^–4^	123.94	< 0.0001^*^^*^^*^
B^2^	8.653 × 10^–3^	1	8.653 × 10^–3^	113.61	< 0.0001^*^^*^^*^
C^2^	9.012 × 10^–3^	1	9.012 × 10^–3^	118.32	< 0.0001^*^^*^^*^
D^2^	8.477 × 10^–3^	1	8.477 × 10^–3^	111.29	< 0.0001^*^^*^^*^
Residual	1.066 × 10^–3^	14	7.617 × 10^–3^		
Lack of fit	8.316 × 10^–4^	10	8.316 × 10^–5^	1.42	0.3293
Pure error	2.348 × 10^–4^	4	5.870 × 10^–5^		

^a^***p < 0.001, extremely significant; **p < 0.01, highly significant; *p < 0.05, significant.

**FIGURE 2:**
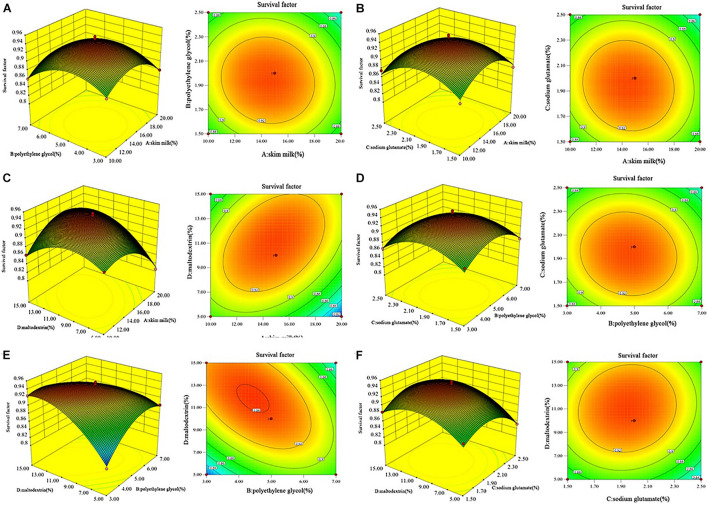
Response surface and contour plots describing the interactive effects of **(A)** skim milk + polyethylene glycol, **(B)** skim milk + sodium glutamate, **(C)** skim milk + maltodextrin, **(D)** polyethylene glycol + sodium glutamate, **(E)** polyethylene glycol + maltodextrin, and **(F)** sodium glutamate + maltodextrin on the survival factor of the artificial starter culture.

The effects of the tested factors on the survival factor were visualized in the response surfaces (Figure 2). The interaction terms of the concentrations of skim milk, polyethylene glycol, sodium glutamate, and maltodextrin demonstrated statistical significance (*p* < 0.0001). The survival factor peaked at 0.942 when the concentrations of skim milk, polyethylene glycol, sodium glutamate, and maltodextrin were 15.09, 4.45, 1.96, and 11.81%, respectively. Using this combination, the survival factor increased significantly by approximately 10% relative to the use of single protective agents before optimization. This suggests that the established predictive model could effectively predict the survival factor. Hence, an artificial starter was established with the optimal protectant combination of 15.09% skim milk, 4.45% polyethylene glycol, 1.96% sodium glutamate, and 11.81% maltodextrin. To further verify the predictive value of the response surface optimization, three repeat experiments were performed using the optimal protectant combination. The average value of the survival factor of the artificial starter was 0.931, and the fitting rate of the predicted value was 98.83%, indicating that the predicted value and the actual value had a good fit. Together, these data suggest that the determined optimal protectant composition can significantly improve the survival factor of the artificial starter.

### Fermentation Activity Analysis of the CRW Samples Brewed With Different Starters

**FIGURE 3:**
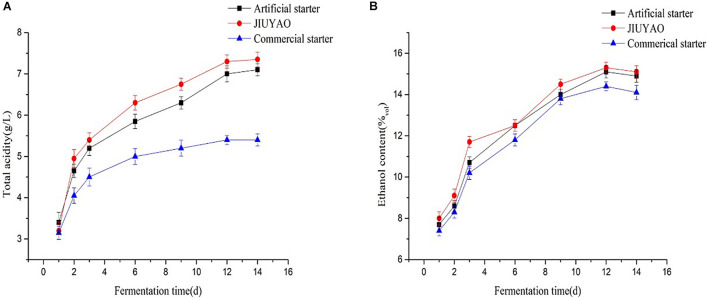
Fermentation performance results of the three Chinese rice wine samples fermented with three different starter cultures. **(A)** Changes in the total acid contents during the fermentation process. **(B)** Changes in the ethanol contents during the fermentation process.

We compared the fermentation activities of the three CRW samples, each brewed with either traditional JIUYAO, the commercial starter, or the artificial starter prepared with the optimal lyoprotectant composition obtained by the response surface method. Saccharification capacity is a key factor that significantly influences wine fermentation (Yao et al., 2018). JIUYAO showed the highest saccharification capacity at 290 ± 3.2 mg/g h, followed by the artificial starter (275 ± 5.3 mg/g h), while the commercial starter showed the lowest capacity (200 ± 4.6 mg/g h). The fermentation activity (ethanol and acid content) of the three CRW samples during fermentation is shown in Figure 3. As CRW is mostly fermented in an open environment, rapid growth of the yeast strains in the starter is required to produce ethanol at the initial stage to inhibit bacterial overgrowth and avoid spoilage (Wu et al., 2015; Yang et al., 2018). The changes in ethanol content during fermentation were similar across the three samples, suggesting comparable ethanol production capacities of all three starter cultures. The total acidity of the wines brewed with JIUYAO and the artificial starter were similar, and were higher than that of the wine brewed with the commercial starter throughout the whole fermentation process. These results suggest that the fermentation activity of the CRW sample fermented with the artificial starter was comparable to that of the sample fermented with JIUYAO, but more vigorous than that of the sample fermented with the commercial starter.

### Electronic Nose Measurements

**FIGURE 4:**
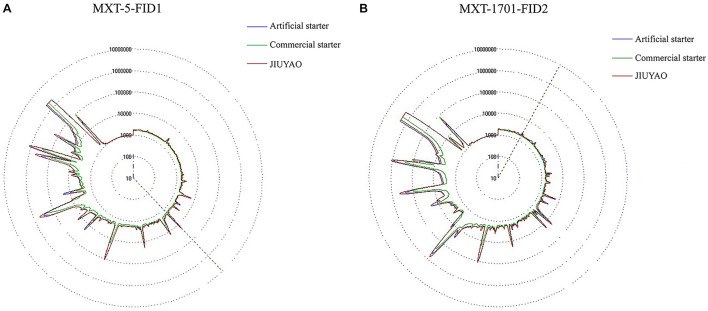
Flavor profile radar chart of three Chinese rice wine samples brewed with three different starter cultures. **(A)** MXT-5-FID, **(B)** MXT-1701-FID.

**FIGURE 5:**
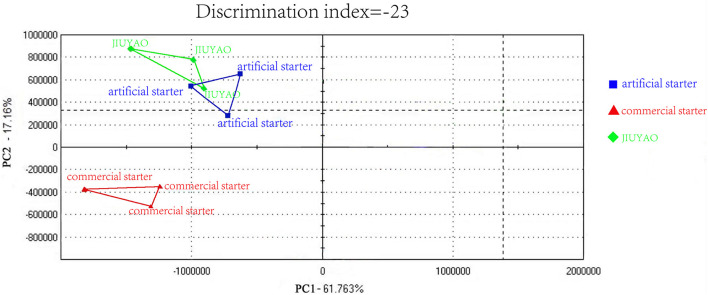
Principal component analysis of the three Chinese rice wine samples brewed with three different starters.

Combining the high-efficiency separation ability of GC and the biological simulation of the sense of smell, the electronic nose can provide a comprehensive aroma profile of volatile flavor compounds (Wu et al., 2012). As shown in Figure 4, the flavor profile radar chart of the CRW samples brewed with the three different starters displays the data intuitively. The overall aroma peak appearance of the samples brewed with JIUYAO and the artificial starter is relatively similar, whereas the peak areas are reduced at multiple positions for the sample fermented with the commercial starter, indicating an overall reduction in aroma intensity. To enable better data discrimination between the three samples, a principal component analysis was performed to identify patterns associated with their individual components (Figure 5). The principal components of the CRW samples fermented with the artificial starter and JIUYAO were closer and overlapped in the same quadrant, suggesting similar aroma profiles, whereas those of the CRW sample brewed with the commercial starter appeared in a different quadrant. These results indicate similarities in the aroma profiles between the CRW samples brewed with the artificial starter and JIUYAO, but some differences in the CRW sample brewed with the commercial starter. These differences may be ascribed to the microbial species composition. Five core species isolated from JIUYAO formed the functional microbial species of the artificial starter culture, whereas the commercial starter contained only two functional microbial species, namely *S. cerevisiae* and *R. oryzae*.

### Volatile Flavor Compounds of the CRW Samples Fermented With Different Starters

In total, 51 flavor compounds, including 27 esters, 8 alcohols, 1 aldehyde, 4 phenols, 2 ketones, and 5 acids, were detected in the three CRW samples by HS-SPME/GC-MS (Table 3). A total of 50, 39, and 20 aroma compounds were identified in the CRW samples fermented with JIUYAO, the artificial starter, and the commercial starter, respectively.

**TABLE 3:** Relative contents of volatile compounds in the Chinese rice wine samples fermented by different starter cultures (μg/kg, *n* = 3), as identified by HS-SPME/GC-MS.

No	Compound^a^	Identification^b^	RI		Concentration (μg/kg)		
			Calculate	Reference	A^c^	B	C
1	Ethyl Acetate	RI, MS	898	894	570.78 ± 0.17b^d^	613.63 ± 0.96c	325.68 ± 0.46a
2	Ethyl propionate	RI, MS	969	964	25.92 ± 0.07b	28.23 ± 0.65c	11.86 ± 0.16a
3	Isobutyl acetate	RI, MS	1028	1029	11.59 ± 0.32	7.65 ± 0.16	–^d^
4	Ethyl butyrate	RI, MS	1031	1039	81.71 ± 0.08c	63.16 ± 0.28b	45.29 ± 0.15a
5	Propanoic acid, pentyl ester	MS	1064	–	20.44 ± 0.64	–	–
6	Isobutanol	RI, MS	1086	1092	377.91 ± 0.26c	161.82 ± 0.48b	98.62 ± 0.12a
7	Isoamyl acetate	RI, MS	1119	1126	464.08 ± 0.15	116.15 ± 0.71	–
8	1-Butanol, 3- methyl-, acetate	RI, MS	1135	1129	416.49 ± 0.15b	596.09 ± 0.61c	348.98 ± 0.54a
9	Pentanoic acid, ethyl ester	RI, MS	1140	1142	8.78 ± 0.41	–	62.38 ± 0.48
10	isopentanol	RI, MS	1200	1206	4459.53 ± 10.14	860.82 ± 2.14	–
11	2-methylbutanol	RI, MS	1205	1208	3527.85 ± 0.16	338.94 ± 0.12	–
12	2-Pentylfuran	RI, MS	1234	1235	26.57 ± 0.54	4.62 ± 0.01	–
13	ethyl hexanoate	RI, MS	1238	1241	185.64 ± 0.15a	245.98 ± 0.77b	556.31 ± 0.84c
14	Acetic acid, hexyl ester	RI, MS	1270	1276	14.93 ± 0.18	–	–
15	Ethyl heptanoate	RI, MS	1332	1332	18.24 ± 0.20	3.41 ± 0.02	–
16	1-Hexanol	RI, MS	1365	1361	73.26 ± 0.33	1.8 ± 0.08	–
17	1-Propanol, 3-ethoxy-	RI, MS	1358	1359	65.46 ± 0.34	5.06 ± 0.84	–
18	2-Nonanone	RI, MS	1395	1398	4.65 ± 0.22	–	–
19	acetic acid	RI, MS	1428	1427	82.83 ± 0.15	–	215.89 ± 0.61
20	ethyl caprylate	RI, MS	1436	1441	987.58 ± 0.09	1441.45 ± 1.16	–
21	1-Octen-3-ol	RI, MS	1453	1456	13.53 ± 0.25	29.8 ± 0.21	–
22	furfural	RI, MS	1482	1482	10.42 ± 0.15a	16.08 ± 0.12c	15.56 ± 0.51b
23	1-Hexanol, 2-ethyl-	RI, MS	1502	1484	12.15 ± 0.61	–	–
24	Butanoic acid, 3- hydroxy-, ethyl ester	RI, MS	1516	1522	34.92 ± 0.55	–	–
25	Ethyl nonanoate	RI, MS	1540	1541	84.28 ± 0.40	20.73 ± 0.05	–
26	n-Caprylic acid isobutyl ester	RI, MS	1557	1561	7.59 ± 0.56	5.02 ± 0.03	–
27	Caryophyllene	RI, MS	1593	1594	24.56 ± 0.27	11.12 ± 0.12	–
28	Ethyl decanoate	RI, MS	1640	1643	1021.13 ± 0.11b	1829.47 ± 2.98c	96.07 ± 0.05a
29	Butanoic acid	RI, MS	1655	1652	18.1 ± 0.31	–	–
30	Octanoic acid, 3-methylbutyl ester	RI, MS	1665	1670	–	15.71 ± 0.17	–
31	Hexanoic acid, 3- hydroxy-, ethyl ester	MS	1673	1673	12.32 ± 0.52	–	–
32	Acetophenone	RI, MS	1695	1693	68.63 ± 0.29	–	45.94 ± 0.14
33	1-Decanol	RI, MS	1743	1748	61.47 ± 0.13	7.96 ± 0.23	–
34	Phenethyl acetate	RI, MS	1800	1825	560.16 ± 0.30	351.1 ± 0.24	–
35	Ethyl laurate	RI, MS	1835	1835	197.88 ± 0.10b	161.24 ± 0.14a	536.96 ± 0.11c
36	Pentadecanoic acid, 3-methylbutyl ester	RI, MS	1862	1863	50.11 ± 0.53	8.19 ± 0.06	–
37	Phenylethyl Alcohol	RI, MS	1936	1935	6686.56 ± 0.06b	12940.7 ± 5.17c	1584.71 ± 0.96a
38	Ethyl Oleate	RI, MS	1988	1986	1230.5 ± 0.36c	58.95 ± 0.15a	629.12 ± 21.28b
39	Phenol, 4-ethyl-2-methoxy-	RI, MS	2030	2032	90.28 ± 0.24	7.16 ± 0.11	–
40	Octanoic acid	RI, MS	2040	2039	171.5 ± 0.44	28.28 ± 0.05	–
41	Tetradecanoic acid, ethyl ester	RI, MS	2053	2043	179.69 ± 0.43b	68.65 ± 0.51a	238.36 ± 1.30c
42	Nonanoic acid	RI, MS	2175	2169	45.58 ± 0.39	–	–
43	2-Methoxy-4-vinylphenol	RI, MS	2196	2194	167.85 ± 0.57	–	–
44	Phenol, 4-ethyl-	RI, MS	2200	2202	115.39 ± 0.38c	100.48 ± 0.14b	54.89 ± 0.19a
45	Hexadecanoic acid, ethyl ester	RI, MS	2243	2243	–	964.73 ± 0.56	7756.55 ± 29.48
46	Benzofuran, 2,3-dihydro-	MS	2246	–	89.197 ± 0.48	6.9 ± 0.47	–
47	Ethyl 9-hexadecenoate	RI, MS	2272	2267	53.46 ± 0.60	20.44 ± 0.58	–
48	n-Decanoic acid	RI, MS	2276	2275	92.57 ± 0.19b	115.34 ± 0.29c	53.68 ± 0.10a
49	Phenol, 2,4-bis(1,1-dimethylethyl)-	RI, MS	2320	2321	72.43 ± 0.28	13.09 ± 0.12	–
50	Octadecanoic acid, ethyl ester	RI, MS	2450	2455	205.45 ± 0.35b	33.51 ± 0.28a	597.01 ± 5.47c
51	Linoleic acid ethyl ester	RI, MS	2514	2521	–	31.57 ± 0.17	589.58 ± 14.39

*^*a*^Aroma compounds detected in the CRW samples.*

*^*b*^Method of identification: MS, mass spectrum comparison using NIST11.L library; RI, retention index in agreement with literature value.*

*^*c*^A, B, C are CRW samples fermented with JIUYAO, artificial starter and commercial starter.*

*^*d*^Values with different letters (a–c) in a row are significantly different using Duncan’s multiple comparison tests (*p* < 0.05).*

*^*e*^Not detected in sample.*

Esters are the most important and common volatile aroma compounds that impart floral and fruity sensory properties to wine (Huang L. et al., 2018), and can be synthesized by yeast and other microorganisms during fermentation (Comuzzo et al., 2006). In the present study, esters formed the largest group of flavor compounds, with 26 and 13 ester compounds detected in the CRW samples fermented with JIUYAO and the artificial starter, respectively. The ester content of the wine fermented with the artificial starter (6685.06 μg/kg) was comparable to that of the wine fermented with JIUYAO (7076.73 μg/kg), but significantly (*p* < 0.05) higher than that of the wine fermented with the commercial starter (4794.15 μg/kg). Notably, isobutyl acetate, n-caprylic acid isobutyl ester, ethyl heptanoate, ethyl nonanoate, phenethyl acetate, and ethyl caprylate were the only esters identified in the CRW samples brewed with JIUYAO and the artificial starter. Thus, these ester compounds may be significantly correlated with the core microbial species used in the artificial starter.

Alcohols formed the second largest category of flavor compounds in the CRW samples, and are known to be the key aroma components of wine, especially brewed rice wine (Wang et al., 2014). Alcohols are produced through the metabolism of sugars and the decarboxylation and dehydrogenation of amino acids (Hernandez-Orte et al., 2008). Nine, eight, and two alcohol compounds were detected in the CRW samples fermented with JIUYAO, the artificial starter, and the commercial starter, respectively. Only two alcohols, namely isobutanol and phenylethyl alcohol, were identified in the wine fermented with the commercial starter. The alcohol content of the CRW samples fermented with JIUYAO and the artificial starter was 15,212.26 and 14,341.84 μg/kg, respectively, but that of the sample fermented with the commercial starter was only 1683.33 μg/kg. These results indicate that the core microorganisms used in the artificial starter could produce considerably more alcohol compounds during CRW fermentation.

To assess the complex olfactory effects of the different aroma compounds, individual OAVs were calculated (Bavcar et al., 2011). As shown in Table 4, 14 volatile compounds with OAVs ≥1 were identified in the three CRW samples, including 8 esters, 4 alcohols, 1 acid, and 1 aldehyde. Twelve aroma compounds with OAVs ≥1 were found in the CRW samples fermented with JIUYAO and the artificial starter, but only five were identified in the sample fermented with the commercial starter. Among these compounds, ethyl caprylate, 1-octen-3-ol, ethyl decanoate, ethyl butyrate, furfural, isoamyl alcohol, ethyl propionate, isobutanol, and n-decanoic acid were detected only in the samples brewed with JIUYAO and the artificial starter. These compounds contribute to the pleasant aroma profile of CRW due to their desirable aroma and low odor threshold (Yu et al., 2019a). For example, ethyl butyrate imparts an apple-like aroma to rice wine. Isoamyl alcohol is a powerful aroma agent with a banana flavor, which can improve the taste of wine by reducing the bitter-tasting amino acids (leucine) (Chen Z. et al., 2015). These results, together with those of the electronic nose analysis, indicate that the flavor profile—especially the key aroma compounds—of the CRW fermented with the artificial starter is similar to that of the CRW fermented with JIUYAO. Thus, the artificial starter is a potential substitute to JIUYAO to aid the industrial production of high-quality CRW with a stable flavor profile.

**TABLE 4:** OAVs of volatile compounds detected in the three Chinese rice wine samples fermented with three different starter cultures.

No	Compounds	A^a^	B	C	Threshold^c^ (μg/kg)
1	Phenylethyl alcohol	111	216	26	60
2	Ethyl caprylate	5	7	–^b^	200
3	Ethyldecanoate	2	3	<1	530
4	Isoamyl acetate	208	298	174	2
5	1-octen-3-ol	5	11	–	2.7
6	Ethyl hexanoate	<1	<1	1	530
7	Ethyl butyrate	1	1	<1	59
8	Ethyl propionate	1	1	<1	29
9	Ethyl acetate	114	123	65	5
10	Furfural	1	2	–	8
11	Ethyl laurate	<1	<1	1	500
12	Isoamyl alcohol	782	689	–	6.1
13	Isobutanol	12	5	–	33
14	n-Decanoic acid	1	2	<1	70

*^*a*^A, B, C are CRW samples fermented with JIUYAO, the artificial starter and the commercial starter.*

*^*b*^The OAV was not calculated in the sample.*

*^*c*^The detection threshold was drawn from the literature (Van Gemert, 2003).*

## Conclusion

An artificial starter was prepared for high-efficiency industrial CRW production, and the fermentation activities and flavor profiles of CRW samples fermented with JIUYAO, a commercial starter culture, and our artificial starter culture were compared. The optimal lyoprotectant combination was determined as 15.09% skim milk, 4.45% polyethylene glycol, 1.96% sodium glutamate, and 11.81% maltodextrin, using the response surface optimization method. These three different starters had equivalent fermentation activity in terms of the alcohol content of the fermented CRW samples. The sample brewed with the artificial starter showed similar acid content and volatile compound profiles to those of the sample brewed with JIUYAO. Although the aroma compound content of the CRW sample fermented with the artificial starter was lower than that of the sample fermented with JIUYAO, the same main aroma compounds, such as isoamyl acetate, ethyl acetate, phenyl alcohol, and isoamyl alcohol (OAV ≥ 1), were found in both and contributed highly to the flavor of the CRW. Our artificial starter culture is a promising substitute to traditional JIUYAO to aid the industrial production of high-quality CRW with a stable flavor profile. Further studies should be devoted to in-depth analysis of the associations between the core microbes and flavor substances to enhance the activity and stability of the artificial starter during batch production of CRW.

## Data Availability Statement

The original contributions presented in the study are included in the article/supplementary material, further inquiries can be directed to the corresponding author/s.

## Author Contributions

CC wrote the manuscript and performed the statistical analyses. ZL performed the statistical analyses and flash GC electronic nose detection. WZ compared the fermentation activities between different samples. HT determined the flavor profiles of samples by gas chromatography–mass spectrometry. JH and HYua established the optimal protectant combination for lyophilization of the artificial starter. HYu designed the research. All authors contributed to the article and approved the submitted version.

## Conflict of Interest

The authors declare that the research was conducted in the absence of any commercial or financial relationships that could be construed as a potential conflict of interest.

## Publisher’s Note

All claims expressed in this article are solely those of the authors and do not necessarily represent those of their affiliated organizations, or those of the publisher, the editors and the reviewers. Any product that may be evaluated in this article, or claim that may be made by its manufacturer, is not guaranteed or endorsed by the publisher.
